# A biodiversity monitoring program for macroinvertebrates inhabiting streams and rivers of South Tyrol (Italy): aims, methodologies, and publicly accessible dataset

**DOI:** 10.1016/j.dib.2022.108648

**Published:** 2022-09-30

**Authors:** Alberto Scotti, Thomas Marsoner, Magdalena Vanek

**Affiliations:** Institute for Alpine Environment^2^, EURAC Research, Drususallee 1, Bozen 39100, Italy

**Keywords:** Alps, Bioassessment, Benthic macroinvertebrates, Geomorphology, Nutrients, Running waters, GIS, Species, Community

## Abstract

Despite very limited in their extension - about 1% of the total surface of our planet – freshwater habitats greatly contribute to the biodiversity of Earth, since 10% of the known species and 33% of the vertebrates inhabit freshwaters.

However, continuous monitoring of habitats and biodiversity – not only aquatic – is considered as a complex task due to the long-term perspective these monitoring programs should have, and the connected required financial needs.

Here, within the framework of a regional-scale program of biodiversity monitoring started in the mountainous region of the Autonomous Province of Bolzano/Bozen (Italy) – including terrestrial and aquatic habitats – we present a dataset covering the first year of the fieldwork campaign aiming at sampling and identifying the benthic macroinvertebrates inhabiting the running-waters of the region.

First, we developed a GIS model with the aim of classifying all the running-waters of the region on the base of their water origin, elevation, mean discharge, slope, and geology of the catchment. After having identified a final set of 12 different stream types, 10 sampling points per each type were selected throughout the region, 2 of which – defined as “reference points” – were scheduled to be sampled each year, in order to keep a “year-by-year” temporal resolution in addition to a long-term one. Thus, every year, 48 points are sampled: 24 “reference points” (2 sites x 12 stream types), and 24 additional sites whose re-sampling is scheduled to happen every 4 years. In summary, in a 4 year-period all the 120 sites are sampled, and the same sampling campaign is planned to be repeated every 4 years, in order to build an ecological time series in a long-term perspective.

At each site, we collect benthic macroinvertebrates through a kick-net sampler (mesh size 500 µm), following a detailed protocol involving, among other aspects, characterization and quantification of the habitats present in the stretch selected for the sampling, as well as measurement of the water velocity associated to each habitat. The benthic samples are then sorted in the lab – with no application of sub-sampling – and identified mostly to family or genus level using appropriate literature.

In addition, at each site, water samples are collected and analyzed within the same day, through a spectrophotometer, looking for a set of chemical species of nitrogen and phosphorous.

**Specifications Table**

**Table:** 

Subject	Biological sciences -> Biodiversity
Specific subject area	Environmental Monitoring, Freshwater Ecology, Limnology, Biodiversity
Type of data	Text, table
How the data were acquired	In general, the planning and operative phases of the benthic macroinvertebrates sampling campaign were carried out on the base of Stucki et al. [1]. In the field: - kick-net sampler (0,23 × 0,22 m; mesh size 500 µm); - portable probes HI 98198 and HI9829 (Hanna Instruments); - flowmeter “Flowatch” (JDC Electronics); - speditive assessment of stream morphology and channel stability following “Stream reach inventory and channel stability evaluation” by Pfankuch [2]. In the lab: - spectrophotometer DR1900 (Hach Lange); - stereomicroscope (up to 50x magnification); - microscope (up to 400x magnification).
Data format	Raw
Description of data collection	Stream benthic macroinvertebrates were sampled in 48 sampling points across the Autonomous Province of Bolzano/Bozen (Italy). Concurrently, at each point, a set of abiotic parameters describing water quality, stream/river geomorphology and in-stream habitat characteristics was also collected.
Data source location	Autonomous Province of Bolzano/Bozen (South Tyrol), Italy
Data accessibility	Dataset of faunal composition and abiotic parameters is permanently stored by PANGAEA - Data Publisher for Earth and Environmental Science, and it is publicly accessible at this web address: https://doi.pangaea.de/10.1594/PANGAEA.941503
Related research article	

## Value of the Data

• The dataset provides a detailed overview of benthic macroinvertebrate communities inhabiting different habitats of stream/river types widely present across the whole European Alpine Arc. A set of abiotic parameters associated to the macroinvertebrate taxa is also included.
• Given the wide range of information here included – ranging from distribution of benthic macroinvertebrates, to water quality parameters, to geomorphological information – a wide variety of stakeholders (e.g. scientists, practitioners, public authorities) can all benefit from this dataset. In addition, the dataset constitutes a good example of a clearly defined methodology to monitor benthic macroinvertebrates both in the short and long-term, possibly defining a standard for similar programs established around the world.
• This dataset is not only intended to serve as a solid base to pursue basic research for a wide range of ecological research questions – involving e.g. autecology and/or spatial distributions of macroinvertebrate taxa, but it is also intended to provide the scientific basis for local political decisions concerning spatial planning, agriculture and nature conservation: for instance, data may support authorities responsible for decisions concerning renaturation and biodiversity conservation of running-waters.

## 1 Data Description

The dataset includes faunal and abiotic data collected in 48 streams and rivers spread across the territory of the Autonomous Province of Bolzano/Bozen (Italy). These sampling points are part of a regional-scale program of biodiversity monitoring started in the area – including terrestrial and aquatic habitats, see https://biodiversity.eurac.edu – and here we present data covering the first year of the fieldwork campaign aiming at sampling and identifying the benthic macroinvertebrates inhabiting the running-waters of the region.

The faunal data includes the distribution of 186 benthic macroinvertebrate taxa – the total richness retrieved considering all the sampling sites, without resolving taxonomic ambiguities – and their true and calculated density (standardized as individuals/m^2^), also in relation to patch-level information such as the water velocity and substrate that was sampled: mobile blocks, moss, hydrophytes, coarse particulate organic matter (CPOM), big sediments, gravel, helophytes, fine sediments, sand, bedrock and algae.

The total amount of benthic macroinvertebrates collected is 90,063. The greatest amount are identified at family/subfamily (50% of individuals) and genus (42%) level, followed by species level (8%). Most present order is Diptera (52% of individuals), followed by Plecoptera (23%), Ephemeroptera (15%), Oligochaeta (4%), and Trichoptera (3%). The remaining 3% of individuals are part of the orders Crustacea, Coleoptera, Gastropoda, Bivalvia, Tricladida, Hirudinea, Odonata, and Megaloptera.

The abiotic data includes all the environmental parameters that are measured/collected in relation to each sampling sites. More specifically, parameters included are the geomorphological parameters stream width, streambed width, wetted perimeter, and the Pfankuch Stability Index (PSI) scores. In terms of physical and chemical characteristics of water, data available are pH, water temperature [°C], dissolved oxygen (DO) [%, mg/L], oxidation-reduction potential (ORP) [mV], conductivity [µs/cm] and turbidity [FNU] and, as water nutrients, total Nitrogen (TN) [mg/L], total Phosphorus (TP) [mg/L], Orthophosphate (PO_4_) [mg/L], Ammonium (NH_4_) [mg/L], Nitrate (NO_3_) [mg/L], and Nitrite (NO_2_) [mg/L]. The dataset of faunal composition and abiotic parameters is permanently stored by PANGAEA - Data Publisher for Earth and Environmental Science, and it is publicly accessible at this web address: https://doi.pangaea.de/10.1594/PANGAEA.941503.

Faunal nomenclature fully complies with Fauna Europaea standard [3], whilst the operational nomenclature of the dataset (column names) refers - where possible - to the list of Darwin Core terms [4].

## 2 Experimental Design, Materials and Methods

### 2.1 Site Selection

In order to detect the maximum possible biodiversity, sampling sites were selected considering the entire stream and river network of the Autonomous Province of Bolzano/Bozen (South Tyrol), a 100% mountainous region located in the Italian Central Eastern Alps. Since the classification of river types included in the EU Water Framework Directive [5] only considers catchments whose surface is ˃ 10 km^2^, thus excluding a large number of catchments present in the region, we applied a new method mainly based on a protocol developed by the Swiss Federal Office for the Environment [6].

More specifically, 5 geological, topographic, and morphological parameters - water origin, elevation, slope, main geology of the riverbed, discharge (Table 1) - were combined to assign a unique type – and code – to each stream or river segment of the Autonomous Province of Bolzano/Bozen, where a segment is the portion of stream/river where the above-mentioned parameters remain constant through space.

**Table 1:** Parameters and their respective codes and classes used for classifying the whole stream and river network of the Autonomous Province of Bolzano/Bozen.

Parameter	Classes, codes (in brackets) and thresholds			Code position for stream/river type definition
Water origin	glacial (1)	spring (2)	surface water (3)	Xnnnn
Elevation	lowland (1)	montane (2)	alpine (3)	nXnnn
	< 600 m	600 m < x < 1800 m	> 1800 m	
Discharge (average annual)	small (1)	medium (2)	high (3)	nnXnn
	< 0.05 m³/s*	0.05 < x < 1 m³/s	> 1 m³/s	
Slope	flat (1)	medium (2)	steep (3)	nnnXn
	< 0.5%	0.5 < x < 5%	> 5%	
Geology	carbonate (1)	silicate (2)		nnnnX

⁎ streams with estimated annual discharge < 0.02 m^3^/s were excluded.

All parameters were derived trough a GIS analysis and then added to each stream or river segment using ArcGIS 10.3 [7] spatial allocation tools (*intersect* for vector data*, add surface information* for rasters) and geodata from the GeoCatalogo/GeoKatalog of the Autonomous Province of Bolzano/Bozen [8]:

- water origin was determined by identifying rivers which originate directly from or near glaciers or which start near one or multiple springs. All other streams were classified as surface water streams;
- elevation was assessed using the Digital Terrain Model (DTM 2.5 m);
- slope raster was created using the slope ArcGIS Function using the DTM 2.5 m;
- for the geology parameter, the shapefile of the geological overview map of the Autonomous Province of Bolzano/Bozen was used and all formations were assigned to “carbonate” or “silicate” based on the correct lithology;
- to estimate the average discharge of each stream segment, the flow accumulation was calculated using an annual water-balance (precipitation – evapotranspiration) raster as input weight. Afterwards, to link this information to the streams, the highest flow accumulation near each stream segment was selected (snap pour point). By multiplying this flow accumulation value with the pixel cell size in m² and dividing it by the number of seconds per year, the average annual discharge in L/s was acquired [9].

**Fig. 1:**
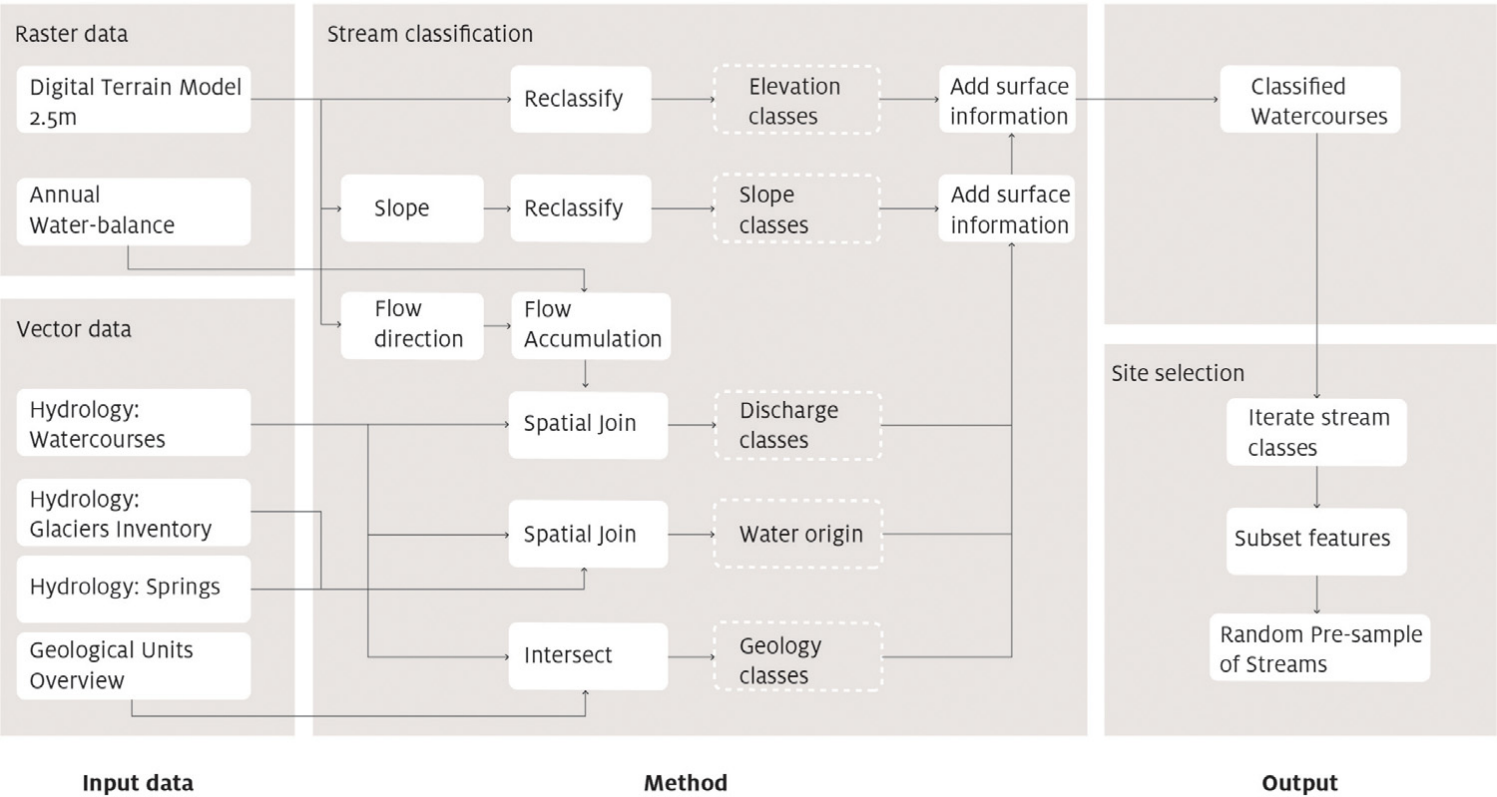
GIS workflow illustrating the process followed from the initial classification of stream and river types of the entire Autonomous Province of Bolzano/Bozen, to the pre-sampling of the final sampling points (© Eurac Research).

The workflow of this analysis is presented in Fig. 1.

**Fig. 2:**
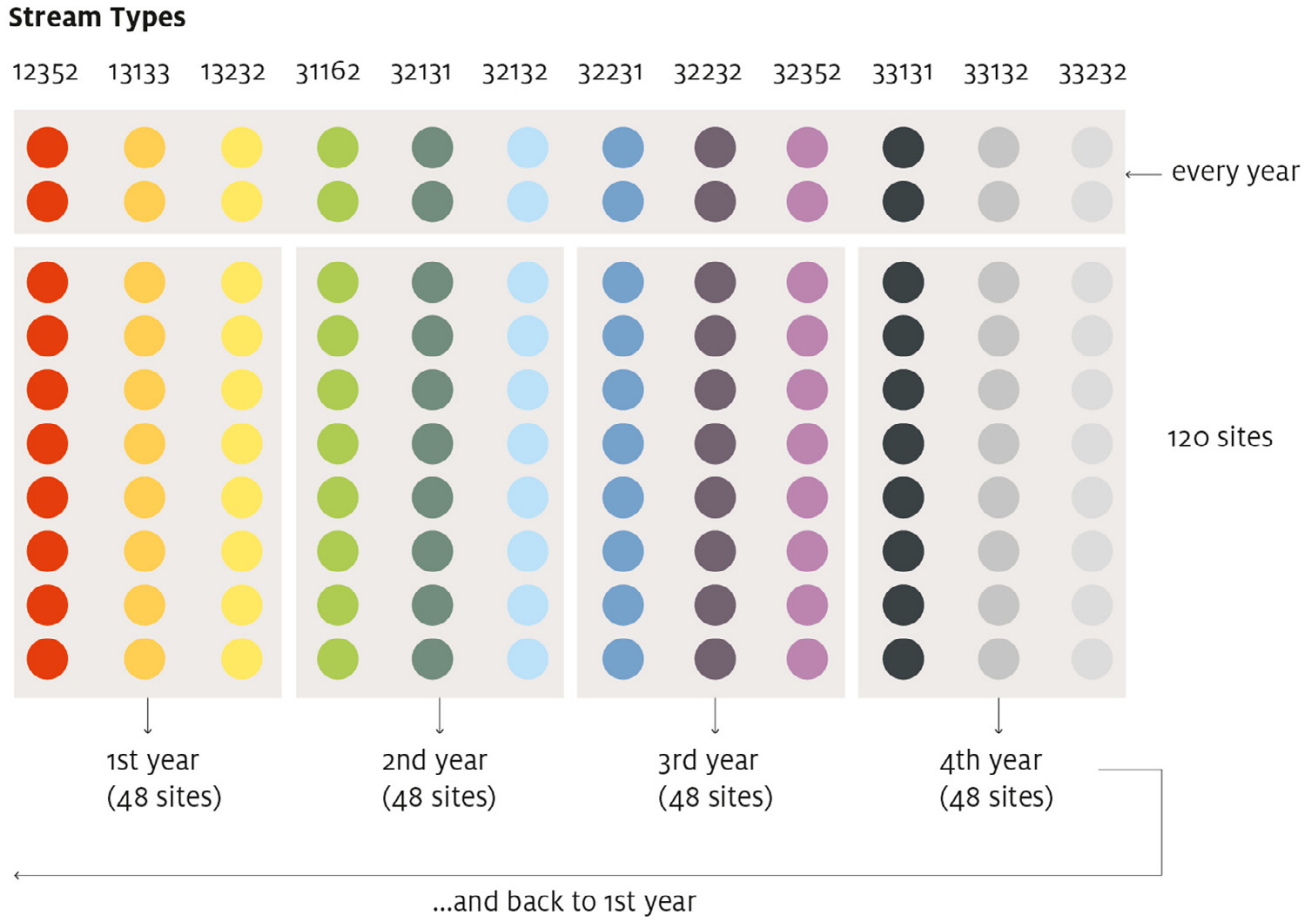
Sampling scheme of the final 120 sampling points constituting the network of the monitoring program. Stream types are here reported in ascending order, but the actual categories that were sampled in full – and included in the dataset described here – during year 1 were codes 31162, 32231, and 32352 (© Eurac Research).

Out of 18 types identified for the region, 12 were retained aggregating some of them. This choice was motivated by the decision of having 10 streams/rivers for each type, in order to have a balanced scheme suitable for consistent statistical analyses (Fig. 2). Merging of classes is tracked by the river type codes since, when the merging happens, the code becomes the sum of the two merged classes: for example, the last number of the stream type 13133, “3”, indicating the geology class, is the result of merging the two geology classes ”carbonate” (code = 1, see Table 1) and “silicate” (code = 2, see Table 1). For the final selection process of the actual potential sampling sites, a pre-sample of 20 streams for each of these 12 types was selected using the ArcGIS function *Subset features* with seed value 1 and random generator type ACM599. Thus, the selected streams were well distributed all over the region, since this function is originally intended to produce a training dataset for a surface model (Fig. 1).

**Fig. 3:**
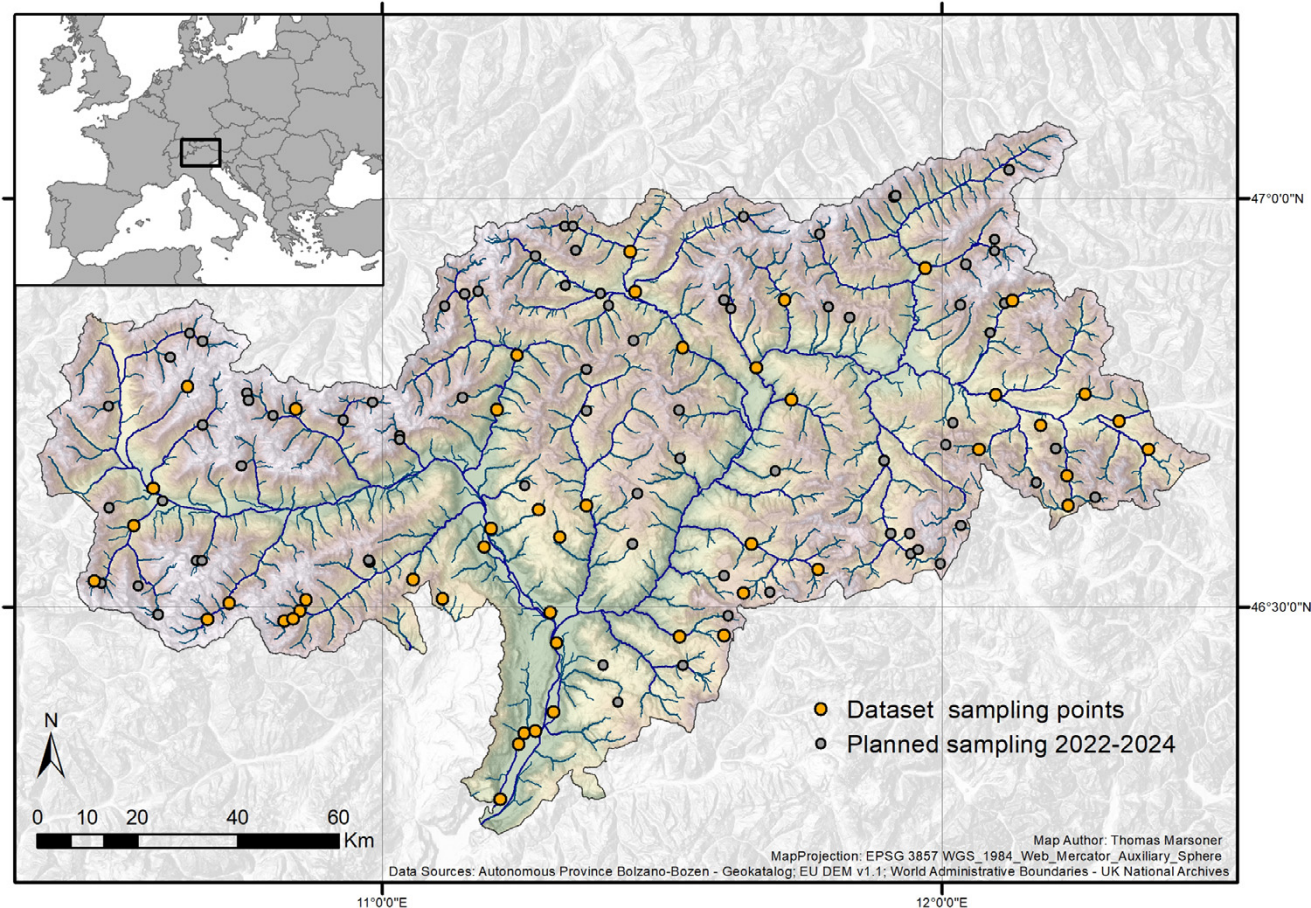
Map of the entire territory of the Autonomous Province of Bolzano/Bozen, showing the locations of the identified sampling sites. Orange ones are the points included in the present dataset, the grey ones are the those that will be sampled in the next 3 years (© Eurac Research).

Out of the 240 randomly selected streams/rivers, the final sampling points selection (10 for each stream/river type) was done manually by visual assessment with orthophotos [8] and having visualized additional accessibility information (e.g. distance and elevation difference to nearest road, distance along a hiking trail to nearest road – accessibility was a prioritization factor). Additionally, nearby locations which are already sampled by the Autonomous Province of Bolzano/Bozen for duties connected to water quality assessments of “EU Water Framework Directive” received a low priority during the selection process. Therefore, in total 120 sampling sites have been identified spread all over the region (Fig. 3).

Every year three entire stream/river type categories are sampled as well as two streams/rivers of every stream/river type (the most geographically apart as possible). Thus, each year 48 sites are sampled (Fig. 2):

• 24 sampling sites are considered as “reference points” for assessing short- term temporal variability, so this subset (the same 24 sampling points) is sampled annually.
• 24 sampling sites are additional points whose re-sampling is scheduled to happen every 4 years, for long-term variability assessments.

In summary, the dataset hereby presented covers the full categories 31162, 32231, 32352, plus 2 “reference points” for all the others. For this dataset (year n. 1 of the biodiversity monitoring program of running-waters of the Autonomous Province of Bolzano/Bozen) faunal and abiotic variables have been collected during year 2021, from March to July (Fig. 3).

### 2.2 Sampling Windows

A standardized framework concerning the “temporal-windows” for the sampling events is applied, in order to have comparability of the monitoring results through years and elevational gradients (see Table 2, adapted from Stucki et al. [1]): applying this method, even at different elevations, climatic and hydrological conditions as well as macroinvertebrate phenology are always comparable within and among years. The most stable hydrological and meteorological conditions are chosen for planning the sampling events. The sampling of the 24 “reference points” preferably takes place during the same months every year, to allow further comparability of the results.

**Table 2.** Sampling windows for benthic macroinvertebrates, as indicated by Stucki et al. [1]. Each month is divided in two different windows, where “I” indicates the ideal period for sampling, and “F” a facultative period in case, for example, of unexpected meteorological or hydrological conditions happening during the “ideal” time frame [1].

	February		March		April		May		June		July	
200–600 m		F	I	I	F							
601–1000 m				F	I	I	F					
1001–1400 m					F	I	I	F				
1401–1800 m						F	I	I	F			
> 1800 m								F	I	I	F	

### 2.3 Fieldwork and Lab Activities

The methodology for the field surveys of benthic macroinvertebrates is based on the Swiss protocol “Methoden zur Untersuchung und Beurteilung der Fliessgewässer: Makrozoobenthos” [1].

**Fig. 4:**
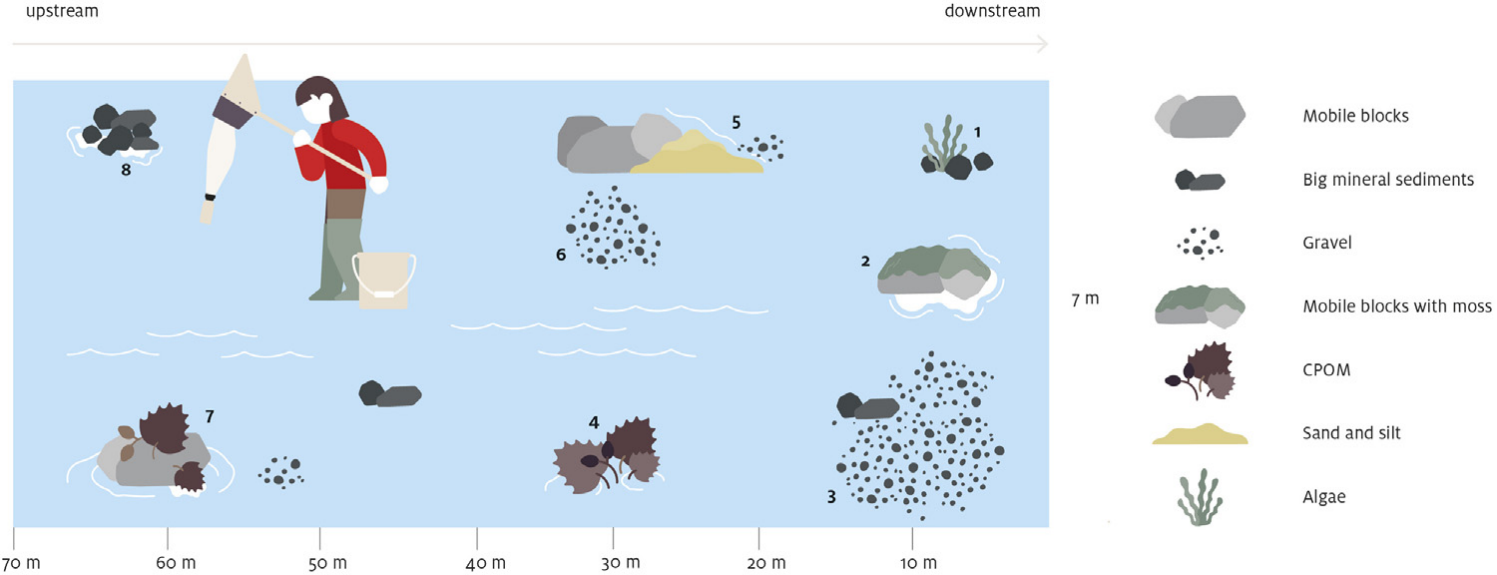
Graphical representation of the sampling method applied for sampling benthic macroinvertebrates. Further details can be found in the text, or referring to Stucki et al. (2019) (© Eurac Research).

The sampling area roughly corresponds to the width of the wetted perimeter x 10 (Fig. 4). Total stream width [m] is measured, however in case of low discharge and/or limited wetted area (e.g. for streams located in the “carbonatic” geologic area) both the streambed (width to bank top) and the wetted perimeter (mid-channel width) were measured. This allows to estimate the percentage of the different substrate types in the sampling area (only substrates covering more than 1% of the sampling are considered), where 8 plots (i.e. subsamples) are taken [1] (Fig. 4). The different substrate types considered for the sampling are:

- mobile blocks/boulders> 250 mm
- moss: bryophytes
- hydrophytes: submerged spermatophytes
- coarse particulate organic matter: leaves, wood, roots
- big mineral sediments: rocks, pebbles; 250 mm > Ø > 25 mm
- gravel: 25 mm > Ø > 2.5 mm
- helophytes: amphibious spermatophytes
- fine, organic sediments: “mud” Ø < 0.1 mm; puddles close to the shore
- sand and silt: Ø < 2.5 mm fine sediments
- natural & artificial surfaces: bedrock, stone slabs, walls; block > Ø 250 mm
- unsuitable habitats i.e. algae, marl or clay

Together with the substrate sampled, also the water velocity upstream of the substrate is measured. When choosing the 8 different subsampling points, priority is given to sampling the maximum number of different substrates (max. 8 different substrates). In case this is not possible, water velocity is used as a second level of differentiation among samples taken on the same substrate [1]. Thus, 8 subsamples of benthic macroinvertebrates within a stream/river, each one representing a different substrate-water velocity combination, are collected.

Organisms are collected through a kick-net sampler (mesh size 500 µm), perturbating an area of approximately 0.05 m^2^ per each subsample, closely following the protocols described by Stucki et al. [1] (Fig. 4). Sampling windows are chosen according to Table 2. After collection, macroinvertebrates are stored in 75% ethanol.

In the lab, benthic macroinvertebrates are sorted – separating them from organic and inorganic debris, always keeping subsamples separated – and identified *via* microscope (up to 400x magnification) and stereomicroscope (up to 50x magnification) using appropriate scientific literature [10], [11], [12], [13], [14], [15], [16], [17], [18]–19].

Environmental data are acquired *via* the multiprobe HI9829 (Hanna Instruments), measuring pH-value, ORP (oxidation-reduction potential), conductivity, turbidity and temperature directly in the field. Dissolved oxygen is measured with a HI98198 (Hanna Instruments) probe. Additionally, a water sample is collected and, within the same day, a set of supplementary analysis is performed: in the lab, using a portable spectrophotometer (Hach Lange DR1900) chemical species of nitrogen and phosphorus are measured through protocols and reagents issued and produced by Hach Lange (Hach Lange method and reagents: LCK 138 LATON, LCK 349 Phosporous total / Phosphate ortho, LCK 304 Ammonium, LCK 339 Nitrate and LCK 341 Nitrite).

Finally, as additional abiotic parameters, stream morphology and channel stability are visually estimated applying the Pfankuch Stability Index (PSI) scores [2], evaluating upper banks, lower banks and bottom substrate through visual evaluation. With the PSI Index, the resistive capacity of mountain stream channels to the detachment of bed and bank materials can be estimated: low PSI values overall indicate higher stability and low sensitivity to disturbance whereas high PSI values indicate lower stability and higher sensitivity to disturbance. The conditions of the channel stability are therefore ranked as excellent, good, fair, or poor.

## Ethics Statements

Our research does not involve any regulated animals, therefore no detailed ethics statement is needed.

## CRediT authorship contribution statement

**Alberto Scotti:** Conceptualization, Methodology, Validation, Data curation, Writing – original draft, Writing – review & editing, Supervision. **Thomas Marsoner:** Conceptualization, Methodology, Software, Formal analysis, Writing – original draft. **Magdalena Vanek:** Methodology, Validation, Data curation, Writing – original draft, Writing – review & editing.

## Declaration of Competing Interest

The authors declare that they have no known competing financial interests or personal relationships that could have appeared to influence the work reported in this paper.

## Data Availability

Abundances of benthic invertebrates and related environmental variables collected for the Biodiversity Monitoring project of South Tyrol - BMS (Italy) (Original data) (PANGAEA).
